# A methodology for assessing the professional development needs of nurses and midwives in Indonesia: paper 1 of 3

**DOI:** 10.1186/1478-4491-4-8

**Published:** 2006-04-19

**Authors:** Deborah Hennessy, Carolyn Hicks, Aflah Hilan, Yoanna Kawonal

**Affiliations:** 1 School of Health Sciences, University of Birmingham, Birmingham, UK; 2 Independent consultant, Jakarta, Indonesia; 3 Indonesian Nurses Association, Jakarta, Indonesia

## Abstract

**Background:**

Despite recent developments, health care provision in Indonesia remains suboptimal. Difficult terrain, economic crises, endemic diseases and high population numbers, coupled with limited availability of qualified health care professionals, all contribute to poor health status. In a country with a population of 220 million, there are currently an estimated 50 nurses and 26 midwives per 100 000 people. In line with government initiatives, this series of studies was undertaken to establish the training and development needs of nurses and midwives working within a variety of contexts in Indonesia, with the ultimate aim of enhancing care provision within these domains.

**Methods:**

An established, psychometrically valid and reliable training needs instrument was modified for use within the Indonesian context. While this technique has had widespread international use in the developed world, its application for developing countries has not yet been established. The standard form consists of a biographical cover sheet and a core set of 30 items (all health-related tasks), which have to be rated along two seven-point scales. The first of these scales asks respondents to assess how important the task is to their job and the second scale is a self-assessment of respondents' current performance level of the task. By comparing the importance rating with the performance rating, an index of training need can be obtained (high importance and low performance indicating a significant training need). The modifications incorporated for use in this series of studies were a further 10 items, which were constructed following expert group and focus group discussions and a review of the relevant literature. Pilot trials with 109 respondents confirmed its feasibility and acceptability. The instrument was then administered to 524 nurses and 332 midwives across Indonesia.

**Results:**

The data were subjected to a retrospective factor analysis, using a Varimax rotation and Cronbach's α to check the instrument's validity and reliability following modification. The results yielded six factors, which accounted for >53% of the variance, each of which had a Cronbach's α score of between 0.8644 and 0.7068.

**Conclusion:**

The results suggest that the modified instrument remained valid and reliable for use in the Indonesian nursing and midwifery context.

## Background

### Context

Indonesia is a heavily populated, multicultural country, with over 220 million people scattered over 13 000 islands [1]. After the Asian economic crisis in 1998, it was estimated that 80 million people were living below the World Bank poverty line [2]. Internal conflict, together with economic problems, have contributed to a decline in health statistics, particularly in the areas of maternal and infant malnutrition [3], as well as an increased incidence of serious endemic diseases such as malaria, typhoid, cholera, TB [4] and dengue haemorrhagic fever. Consequently, the management of health problems across a cultural and geographically diverse country is a major issue, which has been compounded by the political restructuring in 2001, decentralizing all government departments to autonomous provinces and districts. Embedded in this federalization programme was the requirement, incumbent upon each provincial government, to raise a third of its income independently of central government. A 33% drop in central resourcing represents a significant challenge to health care providers and this, together with the deteriorating heath status of the population, has significantly affected the quality and extent of health care provision.

### Health care in Indonesia

Of the 1000+ hospitals in Indonesia, 66% are publicly funded and the remainder are privately funded. Despite various initiatives, such as Jaringan Pengaman Sosial Bidang Kesehatan, which provides free health care to the poorest sectors, quality of care is variable and health care provision for many disadvantaged groups remains inadequate [4]. Health care overall remains underfunded (2% of the GNP being spent on health in 1990), with the consequence that not only is there inadequate preparation of health care professionals, but service provision is also compromised through lack of equipment and supplies. There is an extensive primary care system, which discharges care through nurse- and midwifery-led, community health care centres, and while largely successful, remote areas remain underresourced. The salience of nursing and midwifery to the discharge of community care is therefore self-evident and it would seem expedient to ensure that the education, preparation and clinical competence of these professional groups is a priority, if health status and care standards are to be improved.

However, the most recent official figures [5] suggest that in 1994, in Indonesia, there were still only 50 nurses and 26 midwives per 100 000 people. While the education and preparation for these roles has increased, in that around 39% of nurses now have diplomas and 1% have degrees, the remaining 60% are educated at secondary school level, which effectively means that teenagers may be undertaking complex midwifery and nursing procedures without the appropriate experience or training. Furthermore, those nurses and midwives educated to diploma and degree level tend to move into education, often upon completion of the qualification, which means that their clinical experience and knowledge of real health care issues are limited.

The educational profile of these professional groups means that clinical standards are often suboptimal, in that the majority of health delivery is undertaken by nurses and midwives educated to secondary school level only, with the higher, more specialist qualifications being delivered by educators with restricted clinical experience. This, together with a context of high demands for health care, restricted resources and limited equipment and facilities, inevitably presents a real challenge for the Indonesian health system. It is fair to say that the position of nursing and midwifery is in stark contrast to that of medicine, which is all-graduate, is considered to be a high-status profession and is the dominant force in the Indonesian health care system. While this is hardly unique, it has the effect of disempowering nurses and midwives, reducing their capacity to instigate change, and thereby further undermining this facet of health care delivery.

### Context-specific problems for nursing and midwifery in Indonesia

The current situation with regard to nursing and midwifery provision in Indonesia is, however, further complicated by a number of factors. First, there is no statutory regulatory authority for nurses and midwives, and consequently there are no regulatory standards for education and clinical competence (although work is in progress to develop these authorities). As already noted, the vast majority of nurses and midwives (60%) have inadequate training and preparation for the role, which creates the potential for substandard care delivery.

Second, there are a number of different grades of each profession, but these are defined inconsistently and the role responsibilities attached to each grade are variably discharged [6]. This problem is compounded by the fact that none of the grades (including those of nursing and midwifery managers) has a job description attached to it [7]. Consequently, it is impossible to monitor the range of work undertaken by these practitioners, its quality or who is discharging the role. The potential for clinical error, with all the associated health risks, then, is high.

Furthermore, the imperative for each government employee in Indonesia to have a satisfactory annual performance review is rendered impossible by the fact that no job descriptions exist against which to measure performance. If the performance reviews are completed, it is often by assessors without nursing or midwifery training, who have little knowledge of the required standards of these groups, nor any objective criteria against which they can be assessed.

### The relationship between education and the delivery of nursing and midwifery services

One consequence of the lack of regulatory standards for education and clinical competence, and the absence of proper job descriptions, is that the level of education and training of many health care professionals does not necessarily match the nature of the work being undertaken. Added to this is that many nurses and midwives have no line of accountability, which engenders a situation whereby practitioners, under pressure from the mounting health demands of the population, may feel obliged to undertake clinical activities that exceed their education or competence level.

While the government is largely aware of these problems and has introduced a number of initiatives in an attempt to improve and maintain standards, it remains the case that many senior health care managers perceive no functional difference between the grades or roles of nurses and midwives, nor between hospital and community settings, even though the problems facing each type of service are quite distinct [7]. An even more extreme view, though one that is widely held, is that the role boundaries of nurses and midwives overlap to the extent that the jobs are interchangeable, with nurses delivering midwifery care and vice versa [7].

In a region where perinatal and maternal mortality rates are high (the former running at 307 per 100 000 live births in 2003 [8] and the percentage of births attended by skilled birth attendants is low (54%), it is clearly essential that properly prepared health care professionals are available to improve neonatal and maternal outcomes [[9], chapter 3]. Indeed, such an objective would be seen to be consistent with Indonesia's Safe Motherhood legislation, primary targets of which are to reduce the number of maternal deaths to 1.5 per 1000 live births, increase antenatal coverage from 51% to 80% and raise the proportion of women with access to safe delivery to 40% [[9], chapter 8].

Indeed, the importance and success of relevant training for midwives in Indonesia has been documented in a number of studies. For example, a programme of training, educating and deploying midwives, which was introduced in remote villages in three districts, had the effect of increasing the number of births attended by skilled midwives from 37% to 59%, and improved midwifery management of obstetric complications [10]. Likewise, an intensive in-service training programme improved the performance of village midwives in three out of five key skill areas [11]. Where qualified midwives have been located in areas of high deprivation in Indonesia, there has been an associated rise in birth weight [12]. These studies and many others confirm the importance of education and training in improving health outcomes.

It is, of course, similarly inappropriate for midwives to discharge nursing duties. Not only are the philosophies of care different, but Indonesia is also experiencing a rise in infectious diseases. Consequently, highly specialized endemic clinical problems, such as dengue haemorrhagic fever, malaria, typhoid, cholera and leptospirosis demand highly specialized clinical care, as well as effective health promotion campaigns [4,13,14].

### The need for regulatory frameworks for nursing and midwifery

Many of the problems in care standards could be addressed through the introduction of formalized regulatory frameworks for training, education and practice for nurses and midwives. Cognizant of this, the Indonesian government and various health groups implemented benchmarking standards for nursing education curricula in 1999, and some community health centres and hospitals have produced formalized competence criteria for nursing and midwifery clinical care. The Ministry of Health and many donors, including the World Health Organization, have produced a large number of clinical standards, guidelines and clinical algorithms (especially where there are no doctors) to support the practice of nurses and midwives.

Although laudable, the dissemination and implementation of these criteria are haphazard, and there are no clear systems to encourage practitioners to alter their practice in line with the recommended guidelines. In reality these standards are seldom found in the clinical sites. Consequently, practice standards continue to be variable and in many cases fall below safe levels. There is widespread criticism of nursing and midwifery care in Indonesia, and yet fundamental issues such as providing care standards and guidelines, defining professional roles and providing in-service training and continuing professional development have not yet been systematically addressed.

### The way forward

Clearly, if standards are to improve, a number of prerequisite actions may need to be considered, namely:

First, it is essential to formulate a definition of each grade of nurse and midwife, in order that performance can be measured against it; this would also enable educational curricula to be devised that accord with the required competence criteria.

Second, it is important that some assessment is made, however rudimentary, of current service delivery levels in nursing and midwifery, so development programmes can be targeted at groups where skill shortfalls are identified.

Third, some evaluation of the education and development needs of nurses and midwives should be undertaken in order to inform in-service training and CPD. Previous training needs analyses in South-East Asia have been scarce and where they have been conducted, have tended to concentrate on local issues, using qualitative methods of observation and interviews [15]. Such approaches are unsuitable for large-scale investigations, which demand a less labour-intensive and more objective focus.

### The aims of the present study

This study, then, was an attempt to provide the information outlined above, in order that:

the role of the nurse and midwife at various grades could be defined, thus providing the baseline framework on which to develop job descriptions;

the current performance levels of these grades of nurses and midwives could be assessed, in order to establish broad parameters of clinical competence and the range of skill levels;

a list of key training needs for various subgroups, such as grade and location of work, could be identified, so that in-service training and continuing professional development programmes could be developed and customized to meet the specific reported needs of each group of nurses and midwives.

This paper is the first in a series of three, which together document the study's methodologies and results. This paper, besides providing the background context for the research, also outlines the methodology used to address the aims outlined above; the following two papers describe the findings for the nursing groups and the midwifery groups.

## Methods

### Design

One of the key requirements in undertaking a large-scale investigation of this type is that the methodology should be cost-effective. Consequently, in-depth interviews, focus groups and other approaches more traditionally associated with a qualitative paradigm are neither practically not financially feasible. Therefore, a self-completion questionnaire survey was adopted. A second key requirement was that the data base should be sufficiently objective and rigorous that it would have the capacity to inform strategy and policy. Therefore, a psychometrically valid and reliable instrument was used that has a well-documented track record for use with health care professionals [16].

### The instrument

Developed in 1996, the main part of the questionnaire consists of a core set of 30 items that can be modified to meet the requirements of a given study without compromising its psychometric properties. All 30 items refer to tasks that are central to the health care professional's role and come within five superordinate categories: research/audit, communication and teamwork, administrative/technical, management/supervisory and clinical. Each item has to be rated along a seven-point scale according to two criteria: how crucial the task is to the successful performance of the respondent's job; and how well the respondent is currently performing the task. The ratings on the first criterion provide an overall occupational profile of the respondent's job (thus addressing the first of the study's objectives), and the second criterion provides an index of skill level or performance (addressing the second of the study's objectives).

A comparison of the two ratings on any item provides an assessment of the training need associated with it, in that tasks considered to be highly crucial but not well-performed have a training implication, while those items for which criticality and performance are rated similarly have little training requirement. This comparison addressed the third of the study's objectives. The training needs can be prioritized in order to identify those in greatest need of development, which is clearly relevant where training and education budgets are restricted. This importance/performance comparison is well established as a means by which training needs can be reliably identified [17]. In addition, the questionnaire has a section for biographical and occupational information, thus affording the possibility of breaking down the sample into different groups for comparison purposes.

The instrument has added benefits in that the method of assessing training needs is relatively opaque and thus reasonably free from deliberate distortion. Standard methods of collecting training needs data tend towards a free-response wish-list approach, which may provide a statement of the respondent's interest and desires rather than reflecting actual skill deficits and areas in need of development [15]. Data triangulation using the instrument has demonstrated a high degree of accord between self-ratings and ratings conducted by others [18].

The instrument has been extensively used with a wide variety of health care professionals. For example, it has been used to identify the training needs of primary care nurses and community and hospital-based nurses [19,20]. It has also been used with allied health care professionals [21,22], in cross-cultural studies of health care practitioners [23,24] and to identify specialist continuing-development needs [19,25,26]. In the majority of these studies, the tool has been modified in order to customize it for the particular aims and objectives of the project; this has not compromised its validity or its reliability [25,27].

However, because the instrument had originally been developed for use in the United Kingdom, it required modification for use in Indonesia. To ascertain the extent of the relevance of the original questionnaire for the current context and to establish what modifications were needed, three sources of information were trawled:

First, a scoping study was undertaken that involved a working party of 21 nurses and midwives, two national consultants and one international consultant, who represented both management and clinical roles. This group met one day a week for six weeks, with the objective of identifying the core tasks and activities that would be expected and required of community- and hospital-based nurses and midwives.

The second source of information that guided the development of the new questionnaire items was a series of semi-structured interviews with key stakeholders at the Ministry of Health, and at health centres in Jakarta, Surabaya and Jombang. The focus of these interviews was the standards, performance and productivity that would be expected of nurses and midwives.

The third data source was a review of relevant studies conducted over the period 1995–2000, which looked at the parameters, level of current practice and possible development needs of nurses.

The information from each of these three activities was distilled via a process of content analysis to yield a number of central themes, and was undertaken by the national working group cited in the first bullet point above. These themes were cross-referenced with the five superordinate categories in the original core questionnaire to check their commonality; any areas not covered by the original instrument were then converted into additional items. In this way, all issues specific to the Indonesian context would be represented. The items were:

applying pharmacology to practice

assessing costs and outcomes of procedures

requesting laboratory investigations and results

consulting with colleagues about care options

developing a shared mission of clinical goals

undertaking budget planning activities

developing joint working arrangements with others

actively assisting in change management activities

locating and accessing relevant equipment for own clinical work.

The high degree of overlap between the themes that emerged from this preparatory activity and the original instrument confirmed its validity for use within the Indonesian nursing and midwifery context; furthermore, the additional items, outlined above, have a great logical appeal, given the nature of the Indonesian health system, reflecting, as they do, the need for a high level of professional autonomy, teamwork and cost-effective health care. Together, then, the resulting items and their method of development add construct, content and face validity to the modified tool.

As with the standard instrument, the modified version had a detailed biographical and occupational section attached to it, so that comparisons of different grades of respondent, area of work and the like could be undertaken. The final instrument, then, consisted of a detailed biographical/occupational section, a 40-item questionnaire and instructions for completion.

The instrument was piloted on 109 respondents (including both nurses and midwives) from Jakarta and West Java with satisfactory results [28]. The only change needed was a clearer guideline for questionnaire completion. The results of the pilot study were presented to stakeholders in the Ministry of Health, the University of Indonesia and the nursing and midwifery professional organizations, who all approved the research methodology. The questionnaire was then administered to 524 nurses and 332 midwives selected from five provinces in Indonesia, covering all professional grades, and representing hospital and community locations. The data from the completed questionnaires were entered into an SPSS database for analysis.

## Results and discussion

Because there were 856 cases and 40 items, the suggested sample size and item:sample ratios were fulfilled [29]. To check the validity of the instrument's structure and its reliability, an exploratory factor analysis was undertaken on the data, following the recommended protocol [29], and using a Varimax (oblique) rotation and subsequent Cronbach's alpha assessment. The factor analysis yielded six factors with component eigenvalues of >1.0, KMO (Kaiser-Meyer-Olkin) measure of sampling adequacy = 0.963 and Bartlett's test of sphericity, p = 0.000. These are presented in Table 1 (factor loadings in brackets).

**Table 1:** Factor structure of modified survey instrument

**Factor 1 (accounting for 36.198% variance, Cronbach's α = 0.8644): Flexibility and application of knowledge**
Actively assisting in change management activities (0.679)
Interpreting own patient data (0.656)
Inputting data into written or computerized records (0.646)
Interpreting results from clinical investigations (0.624)
Planning patients' discharge (0.601)
Instructing/training junior staff (0.583)
Introducing new ideas into own clinical work (0.576)
Locating and accessing relevant equipment for own clinical work (0.448)

**Factor 2 (accounting for 5.144% of the variance; Cronbach's α = 0.8482): Reflective practice**

Appraising own and others' performance (0.610)
Recognizing and managing risk in clinical care (0.594)
Assessing patients' psychological and social needs (0.570)
Consulting with colleagues about care options (0.548)
Critically evaluating published research (0.536)
Assessing patients' physical needs (0.530)
Assisting patients in making informed choices (0.514)
Developing a shared mission of clinical goals (0.466)

**Factor 3 (accounting for 3.428% of the variance; Cronbach's α = 0.8238): *Decision making***

Undertaking health promotion activities (0.628)
Using technical equipment (0.564)
Developing joint working arrangements with others (0.554)
Showing patients and their families how to do things (0.545)
Undertaking budget planning activities (0.535)
Making decisions about patients' clinical problems (0.528)
Working as a member of a team (0.409)

**Factor 4 (accounting for 2.952% of the variance; Cronbach's α = 0.8411): Technical and administrative procedures**

Writing clinical, shift and other reports (0.617)
Collecting own clinical/patient/surveillance data (0.562)
Undertaking technical nursing procedures (0.542)
Getting on with colleagues (0.521)
Prioritizing work according to patients' needs (0.468)
Undertaking clinical examinations of patients (0.454)
Analysing patient data (0.429)

**Factor 5 (accounting for 2.784% of the variance; Cronbach's α = 0.7732): Relationships and investigations**

Identifying areas worthy of investigation in own practice (0.684)
Applying pharmacology to practice (0.678)
Establishing a relationship with patients (0.565)
Designing systems for patient monitoring/observation (0.561)
Liaising with other health care professionals (0.514)

**Factor 6 (accounting for 2.643% of the variance; Cronbach's α = 0.7068): Case management**

Requesting laboratory investigations and results (0.586)
Making appropriate patient referrals (0.549)
Planning/organizing patients' treatments (0.538)
Undertaking administrative duties (0.515)
Assessing costs and outcomes of procedures (0.360)

## Conclusion

The above findings indicate that the instrument, with modifications, remains valid and reliable and therefore suited for use with both nurses and midwives in this study. The final instrument, then, comprised a biographical/educational/occupational section, together with 40 items that had to be rated along a seven-point scale, according to their criticality to the respondent's job, and the current performance level. The customization of the original instrument for use in Indonesia did not compromise its psychometric properties, and its ease of use by the respondents indicated its feasibility as a large-scale survey instrument for use with nurses and midwives in a wide range of health care contexts in Indonesia.

### The expectations of the current studies

It was expected that these studies would provide a baseline measurement of the quality and management of nursing and midwifery across Indonesia. Because Indonesia has a poorer health status than other countries in the region with similar economic resources, it is essential that the quality of the health service and of its health care professionals is monitored and optimized. Concerns about the performance of nurses and midwives, coupled with the absence of any previous evaluative study, prompted the current investigation, the major remit of which was to assess whether the improvement in education from certificate to diploma and degree levels was having any impact on clinical performance.

Furthermore, there were concerns that that Indonesian nurses and midwives would not be acceptable in the international labour market; since one of the labour and international policies of the country is to export more professional staff in order to address the world shortage, this clearly constituted a major setback. The ASEAN countries have a free-trade and mutual recognition agreement for the movement of health care professionals that is due to be implemented shortly. However, since Indonesia has no statutory council for nurses and midwives, there is consequently no recognizable standard by which the quality of health care professionals can be assessed. This series of studies, therefore, aimed to provide information about the current roles of nurses and midwives, from which job descriptions could be developed, clinical skill levels identified and educational/professional development guidelines produced. The information would also be used to generate the framework for a regulatory system that would facilitate the international transfer of health care professionals as well as raising standards of care within Indonesia. The actual implications the studies have had for policy developments are outlined at the end of papers 2 and 3.

## Competing interests

The author(s) declare that they have no competing interests.

## Authors' contributions

DH designed and managed the survey and organized its data collection and preliminary analysis, assisted by AH and YK; CH did a secondary data analysis and wrote the article. All authors read and approved the final article.
